# Operation for Fracture of Spine

**Published:** 1903-11

**Authors:** 


### OPERATION FOR FRACTURE OF SPINE.

John B. Murphy (*Am. Jour. Surg. and Gynecol.*, September, 1903,) says that when there is fracture with primary complete transverse paralysis, operation will do no good.

When there is irregular paralysis, even though it may eventually become complete, recovery usually occurs without operation. When the paralytic symptoms are evidently due to hemorrhage operation is indicated; but it must be done at once. If the paralysis can be ascribed to mere puncture of the cord or to simple pressure of fragments, operation is justifiable; but such cases must be extremely rare.—*Medical Age.*

